# Genetic factors associated with risk of metabolic syndrome and hepatocellular carcinoma

**DOI:** 10.18632/oncotarget.15893

**Published:** 2017-03-04

**Authors:** Ranran Tang, Heng Liu, Yingdi Yuan, Kaipeng Xie, Pengfei Xu, Xiaoyun Liu, Juan Wen

**Affiliations:** ^1^ Nanjing Maternity and Child Health Care Institute, Nanjing Maternity and Child Health Care Hospital, Obstetrics and Gynecology Hospital Affiliated to Nanjing Medical University, Nanjing, China; ^2^ State Key Laboratory of Reproductive Medicine, Nanjing Maternity and Child Health Care Hospital, Obstetrics and Gynecology Hospital Affiliated to Nanjing Medical University, Nanjing, China; ^3^ Department of Pediatrics, Nanjing Maternity and Child Health Care Hospital, Obstetrics and Gynecology Hospital Affiliated to Nanjing Medical University, Nanjing, China; ^4^ Department of Endocrinology, The First Affiliated Hospital of Nanjing Medical University, Nanjing, China

**Keywords:** genetic factors, metabolic syndrome, obesity, HCC

## Abstract

Although the metabolic syndrome is a commonplace topic, its potential threats to public health is a problem that cannot be neglected. As the living conditions improved significantly over the past few years, the morbidity of metabolic syndrome has also steadily risen, and the onset age is becoming younger. The hepatocellular carcinoma (HCC), is one of the most prevalent life-threatening human cancers worldwide, incidence of which is also on the rise, gradually occupied the top of the list associated with metabolic syndrome related complication. Despite the advanced improvement of HCC management, the lifestyle, environmental factors, obesity, hepatitis B virus (HBV) infection have been recognized as risk factors for the development of liver cancer. In recent years, genetic studies, especially the genome-wide association studies (GWASs) were widely performed, a new era of the human genome research was created, which has significantly promoted the study of complex disease genetics. These progresses have contributed to the discovery of abundant number of genomic loci convincingly linked with complex metabolic feature and HCC. In this review, we briefly summarize the association between metabolic syndrome and HCC, focusing on the genetic factors contributed to metabolic syndrome and HCC.

## INTRODUCTION

**Figure 1:**
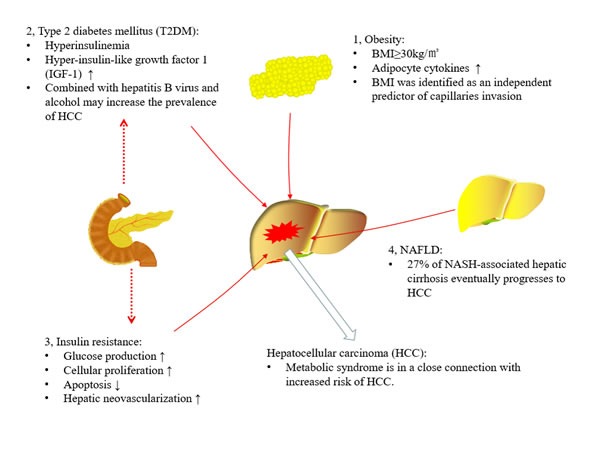
Association between metabolic syndrome and hepatocellular carcinoma (HCC) Metabolic syndrome is in a close connection with an increased risk of HCC. In obese patients, adipocyte cytokines may lead to abnormal lipid profile, and the body mass index (BMI) more than 30 kg/m^2^ could increase HCC risk. In type 2 diabetes mellitus (T2MD), its relative hyperinsulinemia and hyper-insulin-like growth factor 1 (IGF-1) production, or synergistic actions with other variables, such as viral hepatitis and alcohol, all may contribute to the development of HCC. In addition, insulin resistance impaired the ability of insulin to suppress glucose production, and directly accelerate hepatocarcinogenesis via promoting cellular proliferation, inhibiting apoptosis and stimulating hepatic neovascularization. Moreover, bout 27% of non-alcoholic steatohepatitis (NASH)-associated hepatic cirrhosis eventually progresses to HCC.

Metabolic syndrome is a multi-pathological manifestation of syndrome, comprising obesity, dyslipidemia, insulin resistance, and type 2 diabetes mellitus (T2MD), etc. These diseases are in a close connection with increased risk of hepatocellular carcinoma (HCC) occurrence and development (Figure 1) [1]. Based on the existing study, insulin resistance, along with its associated adipocyte cytokines, hyperglycemia, and hyperinsulinemia may lead to hypertension and abnormal lipid profile, both of which will accelerate the progress of HCC [2]. With the wildly performing of genome-wide association studies (GWASs) and gene sequencing, substantial genetic component compositions were proposed [3]. For fat and lean mass in different body regions, including whole body and trunk fat mass, the heritability have been estimated to about 65% [4]. It is supposed that distinction in fat distribution and illustration of genetic factors predisposing to adiposity could contribute to a further exploration of the phenotypic diversity and eventually make a more accurate disease sub-classification possible. In the past few decades, huge efforts have been made to explore the common genetic factors in metabolic syndrome. However, it is a challenging task at last for the extensive heterogeneity. Nevertheless, the pattern which have found common genetic variations associated with various symptoms and diseases, had been broken by the emergence of GWASs [5]. Furthermore, pinpointing genes implicated in metabolic syndrome could be helpful to discover the latent biological pathways, which could be exploited in the development of medical treatment. Here, we briefly summarize the relationship between metabolic syndrome and HCC, focusing on the genetic factors contributed to metabolic syndrome and HCC.

## ASSOCIATION BETWEEN METABOLIC SYNDROME AND HCC

### Obesity and hepatocellular carcinoma (HCC)

Over the past few decades, the prevalence of obesity has sharp increased [6]. Overweight and obesity have been identified as independent risk factors for various cancers including liver cancer, breast cancer, endometrial cancer, colorectal cancer and esophagus cancer. There is also a possible association between obesity and gallbladder, pancreas, thyroid and hematologic malignancies [7]. For HCC, research showed that the body mass index (BMI) more than 30 kg/m^2^ could increase HCC risk [8]. Besides, HCC mortality rates in male with high BMI are 5 times higher than men of normal weight [7]. Comprehensive analysis including 11 studies from Asia, Europe and United States evidenced that both obesity (RR = 1.89) and overweight (RR = 1.17) were closely associated with the development of HCC [9]. SEER-Medicare data analysis showed that adjusted OR of obesity for HCC was 1.93 [10]. The Metabolic Syndrome and Cancer Project (Me-Can) examined 578700 subjects from Austria, Norway and Sweden, showed RR of obesity for the development of HCC was 1.39 [11]. Danish and Korean boffins obtained similar conclusions after analyzing large cohorts of overweight patients [12]. In addition, in a large retrospective cohort including 342 patients who transplanted liver for HCC, BMI was identified as an independent predictor of capillaries invasion [13].

### Type 2 diabetes mellitus (T2DM) and HCC

For developing chronic liver disease and HCC, T2DM act as an independent risk factor [14]. In different cohort studies, the morbidity of HCC among those with diabetes rose from 1.5 to 4 times [10, 15]. A recent meta-analysis of 21 cohort studies demonstrated that the summary RR of HCC with T2DM was 1.86 (95% CI: 1.49-2.31) for patients with chronic liver disease [16]. T2DM combined with its relative hyperinsulinemia and hyper-insulin-like growth factor 1 (IGF-1) production may also accelerate the development of HCC [17]. Diabetes seems to increase the risk of liver cancer by cooperating with other variables, such as viral hepatitis and alcohol [18]. Furthermore, coexistent with diabetes appears to make the HCC relapse rate rise after curative therapy [19].

### Insulin resistance and HCC

Insulin resistance is characterized by reduced sensitivity to insulin and, as a result, impaired ability of insulin to reduce peripheral blood glucose concentration [20]. It is well established that insulin resistance act as a primary dominator that connects all the sections of metabolic syndrome [21]. To overcome insulin resistance and maintain normal metabolic functions, insulin secretion is increased, leading to a state of compensatory hyperinsulinemia [22]. Insulin resistance may directly accelerate hepatocarcinogenesis via promoting cellular proliferation, inhibiting apoptosis and stimulating hepatic neovascularization [23]. However, a 10-year prospective study from Korea of 1,298,385 patients showed that the risk of tumor was influenced by fasting blood glucose concentrations rather than insulin resistance, which challenges the theory that insulin directly effects on promotion of cancers [24].

### Non-alcoholic fatty liver disease (NAFLD) and HCC

NAFLD is the clinical manifestation in liver of metabolic syndrome [6]. In Western countries and Asia, NAFLD is considered as the most common cause of chronic liver disease [25]. The clinic pathological classification of NAFLD include from extensive isolated steatosis (a milder form of hepatic adipose infiltration) to nonalcoholic fatty liver disease (a more severe form of hepatic adipose infiltration). The clinical progress of simple steatosis is relatively gentle, but there are about one third of non-alcoholic steatohepatitis (NASH) affected subjects can develop into liver cirrhosis [26], and about 27% of NASH-associated hepatic cirrhosis eventually progresses to HCC [27, 28]. In recent years, NASH has been considered as an important pathogenic factor of HCC. Thus, since the prevalence of obesity, diabetes and metabolic syndrome continues to rise, a large proportion of the population will have a risk of developing NASH and cirrhosis, and ultimately liver cancer [29, 30].

## SUSCEPTIBILITY LOCI FOR METABOLIC SYNDROME AND HCC

The advances in genome technology, human genome deep sequencing, and the establishment of human variation cataloging, which helped the human genome analysis to create a new era, have further promoted the complex disease genetics research. It was not until 2007 that the genetic map of complex diseases, such as obesity and HCC, had been preliminarily studied by genetic linkage analysis and candidate gene association studies. But for the potential limitations of the design, their application was limited. However, since 2007, through genome-wide association studies, there is a rocket increase in our understandings of specific genetic risk factors for adiposity, insulin resistance, T2MD, NAFLD and HCC. These advances contributed a lot of convincing genetic loci related to the complex metabolic symptom and HCC [31].

### Obesity

The inheritance of obesity has been paid large attention and the genetic research of obesity has been the focus of research. The heritability of obesity is now universally thought to be range from 40% to 70% [32]. In general, as of 2016, GWASs have successfully identified about 400 different loci associated with adiposity phenotypes [http://www.ebi.ac.uk/gwas/]. The discoveries are mainly derived from individual studies and meta-analysis based on BMI as an indicator of obesity. Through GWAS, Frayling et al. reported the first BMI gene locus with significant statistical significance, which is a byproduct of the study for FTO gene associated with T2MD by GWAS [33]. In 2008, a meta-analysis of GWASs from 16,876 European individuals replicated the FTO finding and also found another strong signal 188 kb downstream of the MC4R locus [34]. In 2009, a paper reported a meta-analysis of GWASs from GIANT consortium, which included more than 32,000 individuals with independent replication from >59,000 individuals. They replicated the MC4R and FTO loci and reported six novel loci: TMEM18, SH2B1, GNPDA2, KCTD15, NEGR1, and MTCH2 [35]. In 2012, a meta-analysis of GWASs for BMI in East Asians was performed. They replicated seven previously identified loci (FTO, SEC16B, MC4R, GIPR-QPCTL, ADCY3-DNAJC27, BDNF and MAP2K5) and identified three additional loci in or near the CDKAL1, GP2, and PCSK1 genes [36]. In 2013, Monda et al. conducted a meta-analysis in African individuals and identified two new loci in GALNT10 and MIR148A-NFE2L3 [37]. Then, Pei et al. carried out a meta-analysis of seven GWASs for BMI-related traits from diverse ethnic populations. They confirmed three previously reported obesity susceptibility loci in FTO, MC4R and TMEM18; and identified two novel loci in CTSS and NLK associated with fat body mass [38]. Recently, another meta-analysis among Asians was conducted by Wen et al. and four loci near the KCNQ1, ITIH4, NT5C2 and ALDH2/MYL2 genes were identified [39]. FTO is a common susceptibility gene for BMI in Asian, African and European populations. And it remains the most influential gene, imposing an allelic 0.39 kg/m^2^ increase in BMI [34].

Besides, in 2011, Heid et al. published a meta-analysis of 32 GWASs of Waist-hip ratio (WHR) adjusted for BMI with follow-up of 16 loci in an additional 29 studies. They identified 13 novel loci in or near RSPO3, NFE2L3, VEGFA, TBX15-WARS2, ITPR2-SSPN, GRB14, DNM3-PIGC, HOXC13, ADAMTS9, LY86, ZNRF3-KREMEN1, CPEB4, and NISCH-STAB1 and the known signal at LYPLAL1 unique to fat distribution independently of BMI. In 2013, a GWAS of WHR in up to 27,350 African individuals was performed and rs6931262 at RREB1 was identified for WHR adjusted for BMI [40].

### T2DM

Having a sibling with T2DM will increases the risk of T2DM by two to three times [41], suggesting genetic factors play an important role in T2DM. As of 2016, more than 200 genetic loci have been identified as T2DM risk loci [http://www.ebi.ac.uk/gwas/]. In 2006, the risk variant in TCF7L2 was discovered by a positional linkage strategy in Iceland population [42], which remains the most influential common T2DM variant (allelic OR = ~1.46) [43]. Then it was successfully validated by several other GWASs [44–46]. Soon, multiple T2DM loci were discovered by independent GWASs, such as SLC16A11, CDKAL1, FTO, HHEX, IGF2BP2, KCNJ11, PPARG, SLC30A8 et al.[44, 45, 47]. DIAGRAM conducted the first meta-analysis on T2DM comprising 10,128 European individuals at the discovery stage and identified six novel loci including ADAMTS9, CDC123-CAMK1D, JAZF1, NOTCH2, TSPAN8-LGR5, and THADA [48]. The second meta-analysis by DIAGRAM had a sample size of 8130 cases and 38,987 controls of European descent. Notably 12 T2DM loci harboring genes that included BCL11A, CENTD2, CHCHD9, HMGA2, DUSP9, ZBED3, HNF1A, KLF14, PRC1, KCNQ1, TP53INP1, and ZFAND6 were identified [49]. The third effort made by DIAGRAM included 26,488 cases and 83,964 controls of diverse ethnic populations. Eight novel loci were discovered: ANK1, ANKRD55, BCAR1, CILP2, KLHDC5, MC4R, TLE1 and ZMIZ1 [50]. In addition, AGEN-T2DM conducted a meta-analysis including approximately 55,000 individuals in East Asian populations and identified eight novel T2DM loci in European populations: MAEA, FITM2-R3HDML-HNF4A, GCC1-PAX4, GLIS3, KCNK16, PEPD, PSMD6 and ZFAND3 [51].

Although T2DM and obesity are highly interrelated from both epidemiological and pathophysiological viewpoints, they have few genetic risk loci in common. Of 200 loci associated with T2DM and 400 loci associated with standard measures of adiposity, merely 11 loci are shared (CDKAL1, FTO, GIPR, KCNQ1, LINGO2, LYPLAL1, MC4R, TMEM18, GRB14, RREB1 and ZNF608), which may do not include shared associations below the level of genome-wide statistical significance. However, for SNPs primarily associated with BMI, there seems to be a positive correlation between the effect size on BMI and the effect of the same SNP on T2DM [31].

### Insulin resistance

The T2DM physiologic characteristics are pancreatic β-cell dysfunction and insulin resistance in the peripheral tissues and liver [52]. Interestingly, T2DM susceptibility loci seldom map directly to insulin resistance. Multiple independent studies showed that the insulin resistance susceptibility loci included APOC3, GCKR, IRS1, IGF1, PPARG and so on [53]. By 2016, about 70 genetic loci have been identified as insulin resistance risk loci [http://www.ebi.ac.uk/gwas/]. In 2011, based on the HyperGen study, a GWAS including 1,040 African Americans explored the association between insulin resistance and genetic variation. The results showed SNPs linked with homeostasis model assessment of insulin resistance (HOMA-IR) and fasting insulin near ATP10A (rs6576507 and rs8026527) and CACNA1D (rs1401492) [54].

Obesity is related with insulin resistance, and also a strong risk factor for T2DM. Thus, we can assume reasonably that some loci of obesity would also be insulin resistance loci, or possibly T2DM susceptibility loci. However, there is little overlap among loci for these traits. These results suggest that the etiologically distinct subsets may exist in these extreme phenotypes.

### NAFLD

NAFLD appears family genetic predisposition. Compared with overweight children without NAFLD, fatty liver was found to be more common in children whose siblings and parents had NAFLD. [55]. After adjusting age, race, sex, and BMI, the heritability of NAFLD was estimated to be 39%, suggesting close relationship between the development of NAFLD and genetic factors. To date, a number of potential genetic determinants based on GWASs have been proposed. The Dallas Heart Study firstly identified the nonsynonymous rs738409 (I148M) located in PNPLA3, which was the most important genetic variant associated with NAFLD [56]. After adjustment for ethnicity, BMI, diabetes status and alcohol use, PNPLA3 rs738409 was significantly associated with hepatic fat content. The results were subsequently confirmed by many independent studies [57] and other GWASs [58]. A meta-analysis in 2011 including 16 studies also proposed a strong association between PNPLA3 rs738409 and a more aggressive disease. Homozygous GG will lead to 28% increase in serum ALT levels, 3.5-fold greater risk of NASH, and 3.3-fold higher risk of fibrosis. [59]. The second GWAS about NAFLD was performed in 236 women with NAFLD and identified an association between SNP rs2645424 in FDFT1 (an enzyme with a role in cholesterol synthesis) and NAFLD activity score [60]. In 2011, Speliotes et al. conducted the third NAFLD GWAS [58], and identified five SNPs associated with NAFLD in or near PNPLA3 (rs738408), NCAN (rs2228603), PPP1R3B (rs4240624), GCKR (rs780094) and LYPLAL1 (rs12137855). There was a strong linkage disequilibrium between PNPLA3 rs738408 and the previously identified rs738409 [56]. Another recently GWAS in adolescents with NAFLD identified SNPs relevant to two neuron-specific genes (SLC38A8 and LPPR4) and two liver-specific genes (LCP1and GC). This study further confirmed the significant differential expression of GC and LCP1 in a NAFLD cohort [61]. Another GWAS was conducted in the Japanese population involving 392 NAFLD subjects and 934 controls. In addition to those polymorphisms in the PNPLA3 gene, the polymorphisms in SAMM50 and PARVB were also observed to be associated with the occurrence and progression of NAFLD. [62].

### HCC

As we known, virus infection, obesity, diabetes mellitus, alcohol and aflatoxin B1 exposure are pivotal risk factors for inducing HCC. Genetic factors of the individual genome may also act a role in liver malignant tumor [63]. In recent years, a plethora of studies have confirmed that the host genetic factors play crucial roles in developing HBV-induced HCC. One of the first GWASs included 355 HBV carriers with HCC and 360 asymptomatic HBV carriers (ASCs) in Chinese population, indicated rs17401966 in KIF1B was significantly associated with HBV-related HCC, and SNPs in UBE4B and PGD genes were also shown to be significant for HCC emergence among patients with HBV-positive [64]. In recent years, a large number of studies have confirmed that the host genetic factors played a key role in the development of HBV-related liver cancer. The other two GWASs from Chinese also found some novel SNPs as risk factors for HBV-related HCC, including rs9272105 in HLA-DQA1/DRB1, rs455804 in GRIK1 [65], rs9275319 in HLA-DQ gene and rs7574865 in STAT4 gene [66]. Two GWASs conducted in large Japanese cohorts concluded that variant rs2596542 in the promoter region of the MICA gene [67], and SNP rs1012068 in DEPDC5 gene [68] was significantly related to HCV-induced HCC.

## ANCESTRY-SPECIFIC GENETIC SUSCEPTIBILITY LOCI

Most of the GWASs have been completed in the European, and there are many studies are emerging in other races. Studies of obesity have shown highly comparable effects of common variants across major ancestry groups, strongly supporting shared common BMI and obesity loci across populations, although ancestry-specific loci have also been shown, such as KLHL32 in Africans and KLF9 in Asians [37]. Moreover, of the 14 WHR-associated loci, only 7 were found to have a significant effect in women [69]. For T2DM, studies have reported novel loci such as KCNQ1and C2CD4A in Japanese individuals [70] and a number of loci for T2DM in East Asians [51, 71]. The ancestry-specific genetic susceptibility loci also exist in NAFLD and related cancer, including HCC and pancreatic cancer, suggesting great heterogeneity between the genetic background of different races or populations. It also presented challenges for precise medicine and personalized medicine.

## OUTLOOK

We remain in infancy of the research for potential molecular basis in the HCC development. More specifically, in patients with NASH, the genetic variants and mechanisms that drive the development of HCC remains largely mysterious. Based on the current GWASs, we hope to find new genetic components related to metabolic syndrome and liver cancer progression, yet major efforts are still needed to gain biological knowledge from discoveries.

### Seeking the missing heritability

The past 8 years of genetic discoveries brought about by the GWASs approach have meant a giant leap for genetic research of complex traits, with more than 288 genetic loci shown to associate with metabolic traits. Yet, the major part of the genetic predisposition to these phenotypes remains unaccounted since the proportion of variance explained by genetic risk variants discovered to date is limited. Implicit in the initial design, GWASs-identified variants in metabolic syndrome and related cancer are common (minor allele frequency >5% in the population). There has been much focus on this missing heritability [72]. The rare alleles with large effects are the primary drivers of common disease, which has received renewed attention. The focus is needed to change to study the individual and combined effect of low-frequency and rare variants in metabolic disease through the analysis of data from microarray-based genotyping and wide-genome sequencing.

In addition, influences other than main effects of SNPs may explain parts of the susceptibility for metabolic disease. Preliminary evidence supports the role of copy number variations and gene-environment interactions in obesity, T2DM and HCC, as solid findings have been reported [73, 74], and future large-scale studies may reveal gene-gene interactions.

### Identifying causal variants

GWASs contribute to find the common genetic variants between normal and pathological features, but the major challenge is how to recognize the precise targets of those associations among them. The studies for investigating gene function for the loci from GWASs are needed, including accurate mapping of GWASs signal(s) combined with genetic epidemiology and bioinformatics methods of the integration and optimization of putative functional SNPs, and in vitro and in vivo experimental verification for predicted molecular mechanism to identify the targeted genes [75].

### Risk prediction

As discussed above, common genetic variants unanimously impose modest risk increments on metabolic syndrome and related cancer. Furthermore, combining these variants does not enable prediction these disease/traits [76, 77]. Future studies are needed to reveal further important genetic susceptibility elements. In addition, systemic integration of complex date obtained from other “omics” such as transcriptomics, proteomics and metabolomics, and modeling research for composite effect on the integrate common metabolic phenotype, is projected to be a breakthrough in the research of genetic determinants of metabolic traits. Besides gaining biological knowledge and allowing the identification of at-risk individuals, hopes have been high that knowledge of genetic risk factors would lead to personalized treatment based on the genetic profile. With the developing of the polymorphism risk-scoring algorithm and gene sequencing, it is plausible for identification high-risk groups for prevention and healthcare, and early screening for potential biomarkers. And the complex molecular mechanisms for the NASH, related cirrhosis and HCC need more retrospective and prospective studies to underpin. Since there is a strong association between NASH and HCC, the direction of the study about NAFLD/NASH treatment should focus on how to reduce the risk of cancer in these patients, provide long-term benefits and reduce socio-economic pressure. With the continued popularity of obesity, the prevalence of diabetes mellitus and metabolic syndrome is increasing, so it is imperative to screen the high risk groups of HCC in patients with metabolic syndrome, and to provide the appropriate monitoring strategies.
